# Chromatin differentiation between *Theobroma cacao* L. and *T. grandiflorum* Schum

**DOI:** 10.1590/S1415-47572009005000103

**Published:** 2010-03-01

**Authors:** Liliane G. Dantas, Marcelo Guerra

**Affiliations:** Laboratório de Citogenética Vegetal, Departamento de Botânica, Universidade Federal de Pernambuco, Pernambuco, RecifeBrazil

**Keywords:** cacao, cupuaçu, heterochromatin, karyotype, rDNA sites, *Theobroma*

## Abstract

A comparative analysis of mitotic chromosomes of *Theobroma cacao* (cacao) and *T. grandiflorum* (cupuaçu) was performed aiming to identify cytological differences between the two most important species of this genus. Both species have symmetric karyotypes, with 2*n* = 20 metacentric chromosomes ranging in size from 2.00 to 1.19 μm (cacao) and from 2.21 to 1.15 μm (cupuaçu). The interphase nuclei of both species were of the arreticulate type, displaying up to 20 chromocentres, which were more regularly shaped in cacao than in cupuaçu. Prophase chromosomes of both species were more condensed in the proximal region, sometimes including the whole short arm. Both species exhibited only one pair of terminal heterochromatic bands, positively stained with chromomycin A _3_ , which co-localized with the single 45S rDNA site. Each karyotype displayed a single 5S rDNA site in the proximal region of another chromosome pair. Heterochromatic bands were also observed on the centromeric/pericentromeric regions of all 20 chromosomes of cacao after C-banding followed by Giemsa or DAPI staining, whereas in cupuaçu they were never detected. These data suggest that the chromosomes of both species have been largely conserved and their pericentromeric chromatin is the only citologically differentiated region.

## Introduction

*Theobroma* (Malvaceae) is a tropical genus native to South America, comprising some 22 species (Kennedy, 1995) and having *T. cacao* L. (cacao) as its most important representative. The second most important species in the genus is *T. grandiflorum* Schum. (cupuaçu), native of the Brazilian Amazon. It is largely cultivated and commercialized in Brazil where it is consumed as fruit juice, ice-cream, mousse, etc (Alves *et al.*, 2007). A powder similar to cacao (“cupulate”) can also be obtained from cupuaçu seeds.

Cacao products are widely consumed around the world but large crop plantations are restricted to Brazil, Malaysia and a few countries in West Africa. In spite of its economic value, the cacao tree has received little attention as a crop and almost 70% of the currently cultivated plants have never been submitted to any kind of breeding program (Lockwood, 2003). Nevertheless, in the last 15 years a considerable effort has been put into the molecular mapping and genome sequencing of cacao, aiming to prevent the devastating agricultural effects of fungal diseases, like witches' broom disease (Bennett, 2003).

In most crop plants, karyological analyses have been used to characterize cultivars, to integrate genetic and physical maps, to investigate the origin of hybrids, etc (Jiang and Gill, 2006; Moraes *et al.*, 2007). Nevertheless, relatively little is known about the chromosomes of *Theobroma* species. Analyses based on conventional techniques showed that all *Theobroma* species investigated presented the same diploid number (2*n* = 20) and chromosomes with similar morphology, ranging in size between 0.5 and 2.0 μm (Carleto, 1946; Guerra, 1986; Kennedy, 1995). Besides this apparent chromosome stability, meiotic analyses in some cultivars of *T. cacao* have revealed the occurrence of univalents and several multivalent associations, indicating structural rearrangements (Opeke and Jacob, 1969; Carletto, 1974). Until now, the secondary constriction observed in one chromosome pair is the only chromosome landmark known for cacao (Glicenstein and Fritz, 1989).

In the present study, a detailed comparative analysis of mitotic chromosomes of *T. cacao* and *T. grandiflorum* was performed to improve the karyotypes characterization and to identify possible differences between these two species. Four cytogenetic techniques were used: conventional staining of prophase and metaphase chromosomes, C-banding, staining with the fluorochromes chromomycin A_3_/4'-6-diamidino-2-phenylindole (CMA/DAPI) and fluorescent *in situ* hybridization (FISH). Conventional staining with Giemsa or DAPI has allowed the prophase/prometaphase chromosome differentiation of several species, as rice (Fukui *et al.*, 2000) and cucumber (Koo *et al.*, 2005). C-banding identifies most heterochromatin but does not relate to the base pair composition. The fluorochrome CMA preferentially binds to GC-rich DNA sequences (Hou *et al.*, 2004), whereas DAPI preferentially binds to AT-rich sequences (Kapuscinski, 1995). Chromosome double-staining with CMA/DAPI has allowed the identification of AT- and GC-rich heterochromatin fractions in many plant groups (reviewed by Guerra, 2000). FISH with 5S and 45S rDNA probes has also provided additional markers to distinguish the karyotypes of species or cultivars of several angiosperms (Pedrosa-Harand *et al.*, 2006; Moraes *et al.*, 2007).

## Material and Methods

Commercial seeds of both species were germinated in Petri dishes and cultivated in pots at the Experimental Garden of the Department of Botany. Chromosome preparations were obtained from root tips pretreated with 8-hydroxyquinoline (0.002 M) at 18 °C for 4h30 min, fixed in 3:1 ethanol:acetic acid (v/v) at room temperature for 2-24 h and stored at -20 °C.

Fixed root tips were washed in distilled water, digested for 4-5 h at 37 °C in a mix containing 2% (w/v) cellulase (1 U/mg, Onozuka, Serva) and 20% (v/v) pectinase (625 U/mL, Sigma), incubated in 60% acetic acid for 10 min at room temperature and squashed in a drop of 45% acetic acid. Coverslips were removed by freezing in liquid nitrogen and the slides were briefly stained with a DAPI (2 μg/mL): glycerol (1:1, v/v) solution. The best slides were selected and subsequently destained in ethanol: acetic acid (3:1) for 30 min at room temperature, transferred to absolute ethanol and left overnight at 10 °C, air dried and stored at -20 °C.

Three days-old slides were stained with CMA (0.5 mg/mL, 1 h) and counterstained with DAPI (1 μg/mL, 30 min) (Moraes *et al.*, 2007). Cell images were acquired using a Leica DMLB epifluorescence microscope equipped with a Cohu CCD video camera and the Leica QFISH software. Some slides were destained and stored at -20 °C to be used in the FISH experiments. For the C-banding procedure, two days-old slides were hydrolyzed in 45% acetic acid at 60 °C for 10 min, denatured in a saturated barium hydroxide solution at room temperature for 10 min and incubated in 2x SSC at 60 °C for 120 min (Vanzela and Guerra, 2000). Slides were stained with 2% Giemsa and mounted in Entellan or, alternatively, they were stained with DAPI 1 μg/mL for 30 min and mounted in McIlvaine buffer pH 7.0: glycerol 1:1 (v/v). The images were acquired and the best slides were destained and stored at -20 °C for *in situ* hybridization.

The FISH procedure was based on Jiang *et al.* (1995) with small modifications. R2, a 6.5 kb fragment containing an 18S-5.8S-25S rDNA repeat unit from *Arabidopsis thaliana* (Wanzenböck *et al.*, 1997), and D2, a 500 bp fragment of 5S rDNA obtained from *Lotus japonicus* (Pedrosa-Harand *et al.*, 2006), were used as probes. They were labelled by nick translation with digoxigenin-11-dUTP (Roche) and Cy3-dUTP (GE Healthcare), respectively. The slides were denatured in 70% formamide at 90 °C for 7-10 min. The hybridization mixture, containing 60% formamide (v/v), 2x SSC, 5% dextran sulfate (w/v) and 5 ng/μL of probe, was denatured at 75 °C for 10 min. Each slide received 10 μL of the mix containing the probes. The 45S rDNA probe was detected with a sheep anti-digoxigenin FITC conjugate (Roche) and amplified with a donkey anti-sheep FITC conjugate (Dako). All preparations were counterstained with DAPI (2 μg/mL) and mounted in Vectashield (Vector).

The total chromosome lengths (S) and chromosome arm ratios (AR) were estimated using the Adobe Photoshop CS2 version 9.0. Idiograms based on five metaphases were constructed using the Corel Draw version 11 software. The chromosomes were ordered in the idiograms according to the size of their short arms. The position of the CMA^+^ bands and rDNA sites were additionally indicated.

## Results and Discussion

**Figure 1:**
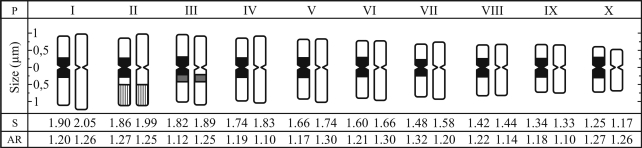
Idiograms of *Theobroma cacao* (left) and *T. grandiflorum* (right). Chromosome pairs (P) are numbered at the top. Chromosome sizes (S) and arm ratios (AR) are indicated at the bottom. Hatched blocks = CMA^+^/45S rDNA; gray blocks = 5S rDNA; black blocks on *T. cacao* chromosomes = C-bands.

The karyotypes were symmetric, consisting of 20 metacentric chromosomes, with arm ratios varying from 1.12 to 1.32 for cacao and from 1.10 to 1.30 for cupuaçu (Figure 1). A chromosome pair bearing a terminal secondary constriction was often observed in metaphases of both species. Glicenstein and Fritz (1989) reported a single satellited bivalent associated to the nucleolus in the meiosis of cacao, but this is the first report of a secondary constrictions in the mitotic chromosomes of cacao and cupuaçu.

The chromosome size ranged from 2.00 to 1.19 μm for cacao and from 2.21 to 1.15 μm for cupuaçu (Figure 1). In previous works, in which the authors analyzed histological sections of untreated root tips (Carletto, 1946, 1971) or squashes of young leaves (Martinson, 1975), slightly different sizes have been reported. The present data, using 8-hydroxyquinoline pretreated root tip cells, suggest that the chromosome size of cacao and cupuaçu display only a very small intra- and interspecific variation.

**Figure 2:**
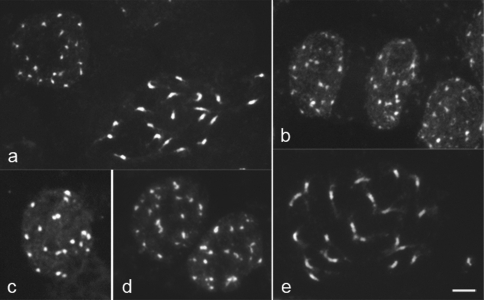
Interphase and prophase cells of cacao (a, c) and cupuaçu (b, d, e) stained with DAPI before (a, b) and after C-banding (c, d, e). Note that the chromocentres are more regular in size and shape in cacao (a, c) than in cupuaçu (b, d). The bar represents 5 μm.

After conventional staining with DAPI, both species displayed interphase nuclei of the arreticulate type, as described previously for cacao by Delay (1949). Cacao nuclei typically exhibited 19-20 chromocentres with regular shape and size, whereas in cupuaçu the chromocentres varied in both shape and size (Figure 2a, b). Conventionally stained and C-banded prophase chromosomes of both species showed a higher condensation in the proximal region and decondensation at one or both chromosome termini (Figure 2a, e), as typically found in species with arreticulate nuclei (Delay, 1949; Guerra, 1987). In general, species with smaller chromosomes tend to display more characteristic arreticulate nuclei (Barlow, 1977; Guerra, 1987), as observed in cacao. However, in this case, the difference in chromosome size between both species is insufficient to explain the different patterns found.

**Figure 3:**
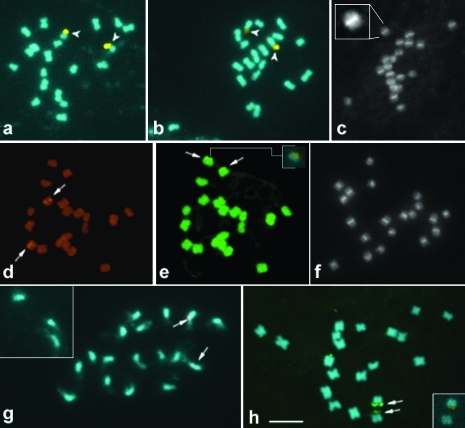
Heterochromatin and rDNA sites in the chromosomes of cacao and cupuaçu. Merged CMA/DAPI metaphase images of cacao (a) and cupuaçu (b). C-banded metaphase of cacao stained with DAPI (c). In the insert, one chromosome in higher magnification. *In situ* hybridization of 5S rDNA (d) and 45S rDNA (e) in cacao.  The insert in (e) shows a CMA+ band co-localized with a 45S rDNA site. Metaphase chromosomes of cacao showing proximal bands after FISH (f). *In situ* hybridization of 5S rDNA (g) and 45S rDNA (h) in cupuaçu. The insert in (g) shows chromosomes that were separated from the metaphase. The insert in (h) showsCMA+ bands co-localized with 45S rDNA sites. Arrowheads and arrows indicateCMA+ bands and rDNA sites, respectively.  The bar represents 5 μm.

In both species, a CMA^+^/DAPI^-^ band was present on the terminal region of the long arm of a single chromosome pair. This CMA^+^ band was frequently heteromorphic in size and distended in one or both homologues. Since most chromosome pairs were similar in morphology and size, it was not possible to precisely determine the position of this pair in the idiogram, although it was probably the second largest pair in both species (Figure 1; Figure 3a, b).

The analysis of the rDNA sites by FISH revealed a single 5S rDNA site in the proximal region of one of the three largest chromosome pairs (Figure 3d, g) and a single terminal 45S rDNA site co-localized with the CMA^+^ band in both species (Figure 3e, h). The 45S rDNA sites exhibited the same size heteromorphism observed with the CMA^+^ bands. Heteromorphism for the 45S rDNA site is usually due to variation in the number of rDNA repeats in each homologue and has been often reported in other genera (see Pedrosa-Harand *et al.*, 2006).

C-banding followed by either Giemsa or DAPI staining revealed 20 well defined chromocentres in interphase nuclei of cacao (Figure 2c) and centromeric or proximal heterochromatic bands of similar size in all its 20 chromosomes (Figure 3c). This heterochromatin distribution should be at least partially responsible for the proximal condensation pattern observed on prophase chromosomes of cacao after conventional staining (Figure 2a). Glicenstein and Fritz (1989) tried unsuccessfully to obtain C-banded chromosomes in cacao using a different technique. In cacao, as in some other genera (Bennett *et al.*, 1995; Vanzela and Guerra, 2000), C-banding differentiation was better when the chromosomes were stained with DAPI than with Giemsa. On the other hand, using the same C-banding technique for cupuaçu, no heterochromatin differentiation was found in metaphase or prophase chromosomes, although the chromocentres were better contrasted after C-banding (Figure 2d) than after conventional staining (Figure 2b). Furthermore, cacao chromosomes often exhibited pericentromeric bands after FISH, equivalent to those revealed by C-banding (Figure 3f), while in cupuaçu such a differentiation was never observed. Analyses of the pericentromeric chromatin in several plant species indicate that this region is prone to accumulate repetitive DNA sequences and rapid differentiation (Lamb *et al.*, 2007). Therefore, the difference between the proximal chromatin of *T. cacao* and of *T. grandiflorum* may be related to the composition of the repetitive DNA sequences of this region. Kawabe and Nasuda (2005) showed that the DNA sequences of the proximal region can change very fast among *Arabidopsis* species which may contribute to speciation. A similar process may have occurred since the beginning of the divergence between the cacao and the cupuaçu genomes. Hybrids between *T. cacao* and *T. grandiflorum* are sterile (Martinson, 1966) and it is possible that the different chromatin organization of their large pericentromeric regions may contribute to constrain the pairing between their homeologous chromosomes.
